# Rare structural variants in the *DOCK8* gene identified in a cohort of 439 patients with neurodevelopmental disorders

**DOI:** 10.1038/s41598-018-27824-0

**Published:** 2018-06-21

**Authors:** Danijela Krgovic, Nadja Kokalj Vokac, Andreja Zagorac, Hojka Gregoric Kumperscak

**Affiliations:** 1 Laboratory of Medical Genetics, University Medical Centre Maribor, Ljubljanska 5, Maribor, Slovenia; 2 Faculty of Medicine, University of Maribor, Taborska 8, Maribor, Slovenia; 3 Department of Paediatrics, University Medical Centre Maribor, Ljubljanska 5, Maribor, Slovenia

## Abstract

Detection of copy number variations (CNVs) is a first-tier clinical diagnostic test for children with neurodevelopmental disorders (NDD), which reveals the genetic cause of the disorder in more than 20%. These are mostly known microdeletion/microduplication syndromes, but variants of unknown clinical significance (VOUS) and ambiguous CNVs can also be detected. An example of the last two are abnormalities in the *DOCK8* gene. Conflicting interpretations of CNVs affecting *DOCK8* can be found in the literature. Deletions were predicted to have a impact in carriers with variable clinical manifestations, where duplications have been proposed as benign variants. In our study, CNV screening was performed in a cohort involving 439 probands with suspected NDD. We identified known microdeletion/microduplication syndromes in 19% and VOUS CNVs in 8% of patients. Among these, three patients had a CNV encompassing the *DOCK8* gene. Although diverse clinical presentations are noted in our three patients, comparison of their phenotypes revealed that abnormalities in cognition and communication, aggressive behaviour and mood swings are common to all of them. Therefore, a clinical relevance, in terms of influencing the psychiatric clinical picture of patients, is proposed for the CNVs disrupting the *DOCK8* gene, regardless of whether it is a deletion or duplication.

## Introduction

The neurodevelopmental disorders (NDD) are a group of conditions with onset in the developmental period. The disorders typically manifest early in development and are characterised by developmental deficits that produce impairments in personal, social, academic or occupational functioning. The range of developmental deficits varies from very specific limitations of learning or control of executive functions to global impairments in social skills or intelligence. According to DSM-5 (Diagnostic and Statistical Manual of Mental Disorders – 5^th^ Edition), NDD include intellectual disability (ID), global developmental delay (DD), communication disorders, autism spectrum disorders (ASD), attention deficit hyperactivity disorder (ADHD), specific learning disorders and other NDD^1^. NDD frequently co-occur; for example, individuals with ASD often have ID/DD and many children with ADHD also have a specific learning disorder.

Molecular karyotyping, which enables the detection of copy number variations (CNVs) in the genome, is a first-tier clinical diagnostic test for children with NDD. In more than 20% of probands, molecular karyotyping reveals the genetic cause of the disorder in terms of known microdeletion and microduplication syndromes^2^. According to the literature, a genetic cause is identified in more than 10% of patients with ASD and in approximately 20% of children with ID/DD^3,4^. No significant CNVs have been linked to ADHD, except in patients in whom ADHD is comorbid with ID/DD or ASD^5^. Most of these genetic abnormalities are well described and their aetiological role is confirmed in many studies. However, in some NDD patients, variants of unknown clinical significance (VOUS) and ambiguous CNVs have also been detected. These are usually rare microdeletions/microduplications encompassing regions that have been rarely or not previously described. Examples of such rare CNVs are *DOCK8* abnormalities on chromosome 9p24.3 - rare small deletions and duplications affecting only the first exon of the gene.

The *DOCK8* gene codes a member of the DOCK family of proteins, which are part of intracellular signalling networks. The product of this gene has an important role in proper immune cell migration, synapse formation and signal transduction^6^. Lack of information in the past, in terms of the gene expression and biological function of *DOCK8*, led to the interpretation of aberrations in this gene, notably duplications, as benign structural genomic variations^7,8^. Only recent reports in the literature of patients with diverse clinical manifestations who harbour these type of aberrations have led to CNVs being considered to have clinical importance^9,10^. In carriers of *DOCK8* deletions, variable clinical manifestations such as ID/DD, facial dysmorphic features, ASD and psychiatric behaviour are usually reported^9–11^. On the contrary, duplications encompassing the *DOCK8* gene are less prominent in the literature^11^.

In this paper, we describe a cohort of 439 patients with NDD who were screened for CNVs. Of these, only three patients had a deletion or duplication encompassing the first exon of the *DOCK8* gene. Since reports of abnormalities in the *DOCK8* gene, especially duplications, are rare and controversial in the literature, we describe the phenotype of these three patients in more detail. Abnormalities in cognition and communication, aggressive behaviour and mood swings are common to all of them. Our findings suggest that CNVs disrupting the *DOCK8* gene have clinical relevance, regardless of whether they are deletions or duplications.

## Methods

### Cohort

A cohort of 439 children with NDD was screened for CNVs in our laboratory between 2011 and 2017. The mean age of included probands was 8.1 years, 300 (68.3%) were boys, 139 (31.7%) were girls. The reason for referral for genetic diagnostic evaluation was suspected NDD according to DSM-5 based on clinical assessment. Genetic analysis was one of the diagnostic tools. If CNVs, which could be associated with NDD, were confirmed, careful further diagnostic evaluation with standardised testing of intellectual and adaptive functions using the Wechsler Intelligence Scale for Children (WISC) for ID and ADOS for ASD, was performed. Probands were classified into three groups according to their primary diagnosis (ASD, ID/DD, and ADHD group). The main characteristics of patients included in this cohort are described in Table 1.

**Table 1:** Characteristics of the patient groups included in the cohort.

Cohort	ASD group	ID/DD group	ADHD group
N = 439	226	183	30
Mean age	7.7 years	5.7 years	11 years
Gender			
Male	173 (76.5%)	102 (55.7%)	25 (83.3%)
Female	53 (23.5%)	81 (44.3%)	5 (16.7%)
CNV detected			
pCNV	31 (14%)	51 (28%)	0 (0%)
VOUS	15 (7%)	12 (7%)	6 (20%)

ASD – Autism Spectrum Disorders; ID – Intellectual Disabilities; DD – Developmental Delay; ADHD – Attention Deficit Hyperactivity Disorder; CNV – Copy Number Variation; pCNV – Pathogenic CNV; VOUS – Variant of Unknown Significance.

Written informed consent was obtained for all probands included in our cohort from their parents or legal guardians and the study was approved by the Commission of the Republic of Slovenia for Medical Ethics (KME No. 89/01/11). All experimental procedures were conducted in line with guidelines and regulations, and abided by the tenets of the Declaration of Helsinki.

The first group is represented by patients with ASD (N 226) with a mean age of 7.7 years. Comorbid mental disorders were present in 43.8% (N 99) from the first group, with 25.7% (N 58) patients having an ID, 9.3% (N 21) a DD, 3.5% (N 8) had combined ID/DD and 4% (N 9) had ADHD as a comorbid disorder. Other symptoms, such as speech delay, dysplastic signs, congenital anomalies and others, were also observed in some patients from the first group. The second group (N 183) comprised patients with ID/DD with a mean age of 5.7 years. Of these, 54.6% (N 100) had DD, and 20.2% (N 37) an ID. ID/DD was observed in 25.1% (N 46) of patients. Additional features, including dysplastic signs, congenital anomalies, speech delay and ADHD, were found in 53% (N 97) patients from group two. The third group (N 30) was comprised of patients with only ADHD. Their mean age was 11 years. Patients with ADHD and ASD or ID/DD as comorbid disorders were included in the first or second group respectively.

The patients with *DOCK8* deletions or duplications were included in the ID/DD group (Patients 1and 2) and the ASD group (Patient 3).

### Patient 1

This girl was a child of non-consanguineous Caucasian parents; her father had been diagnosed with alcohol dependence. She was her mother’s first child; the pregnancy was risky because of insufficient placental development. Her birth was induced in the 8th month of pregnancy. Birth weight was 1880g (60th percentile), length 47 cm (97th percentile) and head circumference 26 cm (3rd percentile). She had microcephaly and palatoschisis. She started to walk at 13 months and had speech problems for which she was treated by a speech therapist. After surgical correction of palatoschisis at the age of 4, her speech improved to some extent, but she still had minor articulation problems. She came to our attention at the age of 15 because of depression, non-suicidal self-injuring (NSSI), aggressive outbursts and the occasional use of psychoactive substances (alcohol, marijuana). She had problems at school, especially in mathematics. According to the Wechsler Intelligence Scale for Children (WISC), her overall level of functioning was average, although an abnormal scatter within the verbal and performance scales was detected and her skills ranged from below to above average. Overall, she managed to adequately sustain her attention, but she became completely confused during the Arithmetic subtest. Her attention span was limited; the results also indicated significant difficulties in working memory. On the D2 Test of Attention, the processing speed was below average, meaning that she solved the tasks slowly, but carefully (rule compliance was average). The quality of performance was average. MRI of the brain was normal as was basic biochemistry, including thyroid hormones and screening for metabolic diseases. Her head circumference was 52 cm (5th percentile). She was diagnosed with a major depressive disorder and NNSI according to DSM-5. She underwent behavioural-cognitive therapy and was started on escitalopram. She is now stable, euthymic, without any NNSI acts, but still abusing alcohol and marijuana occasionally.

### Patient 2

This girl is a twin after *in vitro* fertilisation (IVF) of non-consanguineous Caucasian parents. She was born at 35 weeks gestation with a weight 1800 g (7th percentile) and length 48 cm (48th percentile).

Her twin brother was diagnosed with ASD – Asperger syndrome, at the age of 5. Her mother had suffered from depression with psychotic symptoms at the ages of 17 and 36.

The girl started to walk at 14 months and pronounced her first words at 12 months. At the age of 4, developmental delay and problems with attention and concentration were found. From school entry onwards, she received individual special education help but achieved only minimal school standards.

She was always calm, shy, reserved and quiet and she did not like to be touched, but there was never any suspicion of ASD. She came to our Clinic at the age of 7.5 years on account of her very unusual behaviour, which she had developed in the previous 3 months. She had become confused, her speech had deteriorated, she cried without obvious reason and had undefined fear. On examination, she did not respond to her name, was disorientated and absent, she did not recognise her mother, she talked quietly, her speech being incomprehensible and disorganised. She gave the impression that she had auditory hallucinations. The neurological examination was normal, electroencephalography (EEG) showed irregular and diminished activity with changes in the right centrotemporal region, as seen in rolandic epilepsy. She had never had an epileptic seizure. Magnetic resonance imaging (MRI) of the brain was normal, as well as basic biochemistry, including thyroid hormones and screening for metabolic diseases.

She was diagnosed with a mild intellectual disability and an acute psychotic disorder according to DSM-5. Antipsychotic therapy was suggested, but her mother refused any pharmacological treatment or further diagnostic investigations.

She visited our hospital again at the age of 12 because of an enhanced aggressive outburst, mood swings characterised by euphoric moments, but more commonly, depressive or irritable moods and rage. She had also stopped talking for 4 months and had symptoms similar to the psychotic episode diagnosed at the age of 7.5 years. Most disturbing were the outbursts of aggressive rage, which occurred without any obvious reason and poor reality control, without separating reality from the imaginary world. Repeated MRI of the brain and biochemical tests revealed no pathology and EEG was normal. She was diagnosed with a mild intellectual disability and a recurrent psychotic disorder according to DSM-5. Aripiprazole 5 mg per day was commenced.

### Patient 3

This girl is the only child of non-consanguineous Caucasian healthy parents. She was born 5 days after the expected due date, with weight 3325 g (50th percentile) and length 50 cm (50th percentile). Her developmental milestones were normal and she had no dysmorphisms. Her parents did not notice any abnormalities, but at routine systematic screening at the age of 3 years, developmental delay and problems with articulation were detected. She was treated by a speech therapist and special needs teacher, but she attended a normal school at the special request of her parents. She came to our outpatient clinic because of tension headaches, which were present every afternoon after school, aggressive outbursts, mood changes, tearfulness and stubbornness at the age of 9 years. She presented with impaired social communication, repetitive patterns of behaviour, inflexible order and routine in everyday life and sensory hyperactivity. After a careful diagnostic process, including Autism Diagnostic Observation Schedule (ADOS), she was diagnosed with ASD with a mild to moderate intellectual disability and accompanying language impairment according to DSM-5. MRI of the brain and biochemical tests revealed no pathology. Education at the school for children with special needs was suggested, resulting in cessation of her tension headaches. Sertraline was introduced. She is now stable.

### Genetic analysis

Molecular karyotyping was performed on patients and their parents using DNA extracted from peripheral blood leukocytes with the QIAamp® DNA Blood Midi Kit (QIAGEN, Hilden, Germany). Analysis was performed using BlueGnome CytoChip Oligo (BlueGnome Ltd, Cambridge, United Kingdom) 8 × 60 K and 4 × 180 K arrays following the laboratory protocol and data were analysed using the BlueFuse™ Multi v3.1 software tool (Illumina UK).

### Data availability

All data generated or analysed during this study are included in this published article (and its Supplementary Information files).

## Results

### Genetic studies

We identified a clinically relevant CNV (pathogenic CNV), which could explain the genetic cause of the disorder in 14% (31/226) of ASD patients and in 28% (51/183) of ID/DD patients. There were no pathogenic CNVs in patients in the ADHD group. CNVs with unknown clinical significance (VOUS) were detected in 7% of patients from the ASD (15/226) and ID/DD (12/183) groups, and in 20% (6/30) patients from the ADHD group. Results are summarised in Tables 1 and 2. The exact clinical significance of the VOUS CNVs could not usually be determined due to the unavailability of the patient’s parents or because of the lack of information on the role and function of the genes involved. This latter VOUS group included three patients who were carriers of the genomic alteration affecting the first exon of the *DOCK8* gene, which has an uncertain clinical significance in the literature.

**Table 2:** Summary of the prominent phenotypical characteristics of all three patients.

Clinical features	Patient 1	Patient 2	Patient 3
Birth	risky pregnancy, insufficient placental development, induced, birth weight 1880g (60th percentile), length 47 cm (97th percentile)	IVF, 35 weeks gestation weight 1800g (7th percentile), length 48 cm (48th percentile)	born 5 days postterm, weight 3325 g (50th percentile), length 50 cm (50th percentile)
Dysmorphisms	palatoschisis, microcephaly	no dysmorphisms	no dysmorphisms
Family history	father: alcohol dependence	twin brother: ASD	negative
		mother: depression with psychotic symptoms at age 17 and 36	
Motor development	normal	normal	normal
Cognitive development	discrepant intellectual profile (below to above- average)	mild ID DD at age 4, problems with attention and concentration	mild to moderate ID DD at age 3
Speech	articulation problems/speech sound disorder	normal, periods of mutism	language disorder
DSM-5 diagnosis	major depressive disorder	recurrent psychotic disorder	ASD with mild to moderate ID
Behavioural/mood abnormalities	aggressive, impulsive, violent behaviour, NNSI	aggressive, mood swings from euphoric to depressive, irritable and rage	aggressive, mood changes, tearfulness and stubbornness

IVF – *in vitro* fertilisation; ASD – Autism Spectrum Disorders; ID – Intellectual Disabilities; DD – Developmental Delay; NNSI - non-suicidal self-injury.

The molecular karyotyping analysis indicated duplications in Patient 1 and Patient 3 and a deletion in Patient 2, all involving only the first exon of the longest transcript of the *DOCK8* gene.

The origin of the duplication in Patient 1 was not determined due to the unavailability of both parents, although we excluded inheritance from the mother. Molecular karyotyping in Patient 2 revealed a maternally inherited deletion. The deletion was excluded in her twin brother. The CNV analysis in Patient 3 and both of her parents revealed a *de novo* aberration in our patient. The results of molecular karyotyping for all three patients are presented in Table 3.

**Table 3:** The results of molecular karyotyping for all three patients with CNVs detected in chromosomal region 9p24.3 encompassing the first exon of DOCK8.

Patient	Nomenclature according to ISCN	Size [bp]	Inheritance
Patient 1	arr[GRCh37] 9p24.3(204193_271316) × 3	67,124	/
Patient 2	arr[GRCh37] 9p24.3(204221_266075) × 1	61,855	mat
Patient 3	arr[GRCh37] 9p24.3(204221_271287) × 3	67,067	dn

ISCN – International System for Human Cytogenetic Nomenclature, bp- base pare, mat – maternal, dn – de novo.

## Discussion

An extensive study of CNVs in Slovenian children with NDD confirmed the important role of molecular karyotyping in determining the genetic cause. We identified already known microdeletion or microduplication syndromes as a cause of NDD in 19% (82/439) of our patients (Table 1), which is in accordance with the literature^2^. We also identified a CNV of unknown clinical significance in 8% (33/439) of all patients. Among these, three patients had the rarely identified CNV encompassing the first exon of the *DOCK8* gene. There are contradictory interpretations of its clinical relevance in the literature. Loss-of-function mutations and deletions in the *DOCK8* gene lead to an incomplete immune response, namely the autosomal-recessive form of the hyper-IgE syndrome (HIES)^12,13^. Although *DOCK8* is predominantly expressed in cells involved in the immune response^6^, according to The Human Protein Atlas (https://www.proteinatlas.org/), the protein is also expressed in the brain, mainly in the caudate nucleus, which is engaged for proper cognitive functioning^14^. Apart from HIES, deletions involving the *DOCK8* gene have also been implicated in autosomal dominant ID/DD. The first association of the *DOCK8* gene with ID was established by Griggs *et al*.^15^. Tassano *et al*. further highlighted its role in normal neurological development in their paper, in which two additional patients were reported with a similar interstitial deletion involving the *DOCK8* and *KANK1* genes. Although authors of this report classified deletions as VOUS due to their inheritance from healthy parents and the diverse clinical presentation, the potential role of the *DOCK8* deletion in the irregular neurological development of the patients was proposed^10^.

In our study, Patient 2 was a carrier of a maternally inherited deletion of the *DOCK8* gene. Deletions in this gene have been associated with autosomal dominant mental retardation and DD^15^, which were also present in our Patient 2. Compared to the patients described in the literature and in the DECIPHERv9.21 database (https://decipher.sanger.ac.uk/), with deletions in our region of interest, Patient 2 also shared behavioural problems and mood changes. Our patient had prominent mood swings, ranging from depressive and irritable moods and rage to euphoric moments, but these did not fulfil the diagnostic criteria for bipolar disorder according to DSM-5. Patient 2 had brief psychotic episodes, which have not been described specifically in other carriers with overlapping deletions. Results from the literature support the idea that the deletion in our Patient 2 is probably the cause of the phenotype observed in our patient. Wang *et al*. have already shown in their study that 9p24.3 chromosomal deletions involving the *DOCK8* and *KANK1* genes are a hot spot for CNV loss on chromosome 9. Patients with deletions of *DOCK8* had a strong family history and share the features mentioned above^9^. This was demonstrated in our Patient 2, whose mother had been diagnosed with a recurrent depressive disorder with psychotic features and was a carrier of the same deletion as her daughter. Similar deletions were also presented in the study of Pinto *et al*. on CNVs involved in ASD^11^. Results of our study support the results in the literature suggesting that deletions are clinically relevant.

On the contrary, duplications encompassing the *DOCK8* gene described in patients are less prominent in the literature^11^. According to the population studies gathered in DGV database (dgv.tcag.ca/), the CNVs in *DOCK*8 are not frequently present in the general population, although duplications are more common than deletions. This could be the result of incomplete penetrance, variable expression of the gene and/or the impact of the genetic and epigenetic processes^7,8,10^. Further, there have been several indications in the literature that duplications of the *DOCK8* gene in patients should be considered as benign CNVs, since they were usually inherited from an unaffected parent^7,8^. In fact, Ruiter *et al*. were among the first to classify two smaller duplications in this gene as an innocent variant in probands, although it should not be overlooked that Ruiter’s Case 4 had a behavioural and Case 7 had learning problems, a language disorder and epilepsy^7^. On the other hand, according to Martinez-Jacobo *et al*., 35 patients in the DECIPHER v9.21 database with much larger duplications also encompassing the *DOCK8* gene have psychiatric conditions, a feature they share with the patient from Martinez-Jacobo’s report. They also proposed the association of *DOCK8* with ASD along with the *KANK1* gene^16^. Psychiatric behaviour in association with duplications in *DOCK8* was further described in a study of CNVs as a risk factor for ASD. Oikonomalis *et al*. described a patient with a *de novo* duplication in the *DOCK8* and *KANK1* genes. They also detected a larger *de novo* deletion in the 9p24 region. The smallest critical region in these two patients overlaps the *DOCK8* and *KANK1* genes and was stated to be the region responsible for ASD/ID, since these two features were common to both patients^17^.

Within our study, Patient 1 and Patient 3 had an almost identical duplication and shared a similar clinical picture with regard to cognition, language and psychiatric behaviour. Both had cognitive abnormalities, with an intellectual profile ranging from below to above average in Patient 1 and from mild to moderate ID in Patient 3. They both have a communication disorder, Patient 1 had articulation problems (speech sound disorder) while Patient 3 had a language disorder. Although the core psychiatric diagnoses according to DSM-5 differed (Patient 1 was diagnosed with a major depressive disorder, Patient 3 with ASD), they both presented with aggressive, impulsive and violent behaviour.

**Figure 1:**
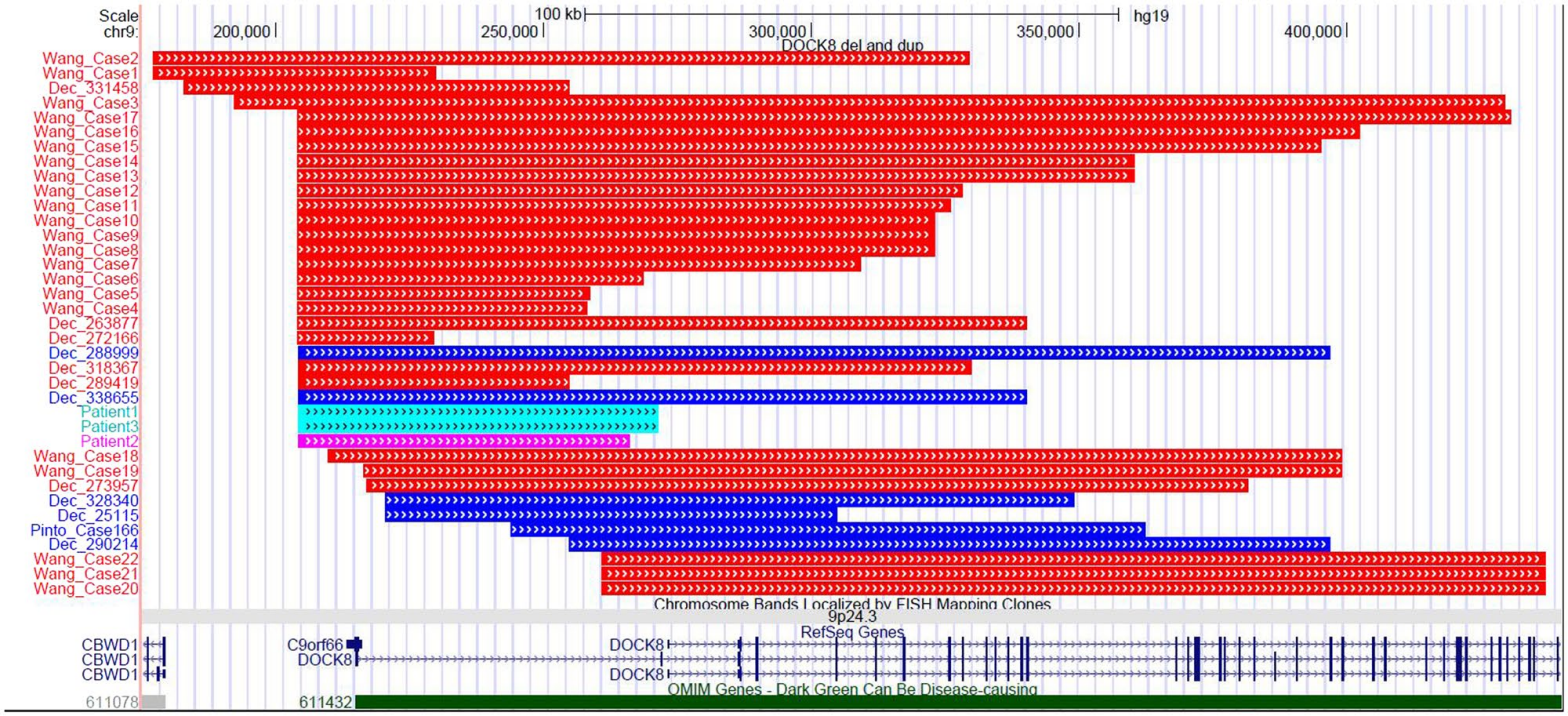
A graphic representation of deletions (red) and duplications (blue) encompassing the first exon of the *DOCK8* described in patients in literature and DECIPHER database, including our three patients (light blue and pink). The exact coordinates and phenotypes (if given) of described patients are listed in Supplemental Table 1. Figure was obtained using UCSC Genome Browser (https://genome.ucsc.edu/).

Our two patients shared clinical features of ASD, ID, DD and aggressive, impulsive, inappropriate and violent behaviour with patients described in the DECIPHER database and Case 166 from the study by Pinto *et al*.^11^ who had duplications coinciding with our region of interest (Fig. 1, Supplemental Table 1). Therefore, in our opinion, the impact of duplications in the *DOCK8* gene on the clinical picture should not be ignored. We therefore propose that *DOCK8* duplications should be at least treated as VOUS and not as benign CNV.

Our extensive study of CNVs in children with NDD confirmed the important role of molecular karyotyping in determining the genetic cause. In agreement with the literature, we identified already known microdeletion or microduplication syndromes in 19% patients in the present study. We also identified 8% CNV with an unknown clinical significance, including three patients with *DOCK8* gene abnormalities. There is increasing evidence in the literature of the importance of *DOCK8* for proper neurological functioning and its influence on some mental disorders and behavioural abnormalities. There are more studies in the literature that support the causative association of the phenotype with *DOCK8* deletions then duplications^10,15^. Deletions were predicted to have a clinical impact in carriers with variable clinical manifestation, the most common being ID, DD, facial dysmorphic features and ASD^9–11^. Duplications, on the other hand, have been proposed as benign variants without any clinical impact, given that they are more common among the general population^7,8^. In the three patients with *DOCK 8* duplications/deletion from our study, a comparison of their phenotype revealed that abnormalities in cognition and communication, aggressive behaviour and mood swings are common to all of them. Hence, our study suggests that CNVs disrupting the *DOCK8* gene, regardless of whether they are deletions or duplications, have a clinical relevance and are associated with a variety of mental disorders/abnormalities.

Considering the limitations of our study, a small number of patients with *DOCK8* duplication/deletion and limited information of gene functions, we strongly suggest further studies on larger number of patients in order to prove the clinical relevance of CNVs effecting the *DOCK8* gene.

## Electronic supplementary material

Dataset 1
